# Prevalence and Epidemiological Determinants of Tinnitus in the Southern Region of Saudi Arabia: A Community-Based Cross-Sectional Study

**DOI:** 10.3390/audiolres16040101

**Published:** 2026-07-10

**Authors:** Abdulrahman Ali Otaif, Musleh Hussain Mubarki, Khalid Talat Ardi, Abdulelah A. Otaif, Dhiyaa A. H. Otayf, Ghala S. Al Ahmari, Danah M. Al Mutib, Razan M. Althumali, Tif A. Jawahi, Najla M. Al Yami, Abdullah M. Algarni

**Affiliations:** 1 Faculty of Medicine, Jazan University, Jazan 45142, Saudi Arabia; 3bdulelahotaif@gmail.com (A.A.O.); dhiyaaot@gmail.com (D.A.H.O.); tifabdu601@gmail.com (T.A.J.); 2 Otorhinolaryngology, Head and Neck Department, Samtah General Hospital, Jazan Region, Jazan 86735, Saudi Arabia; mhmm.1412@hotmail.com; 3 Department of Otorhinolaryngology and Head and Neck Surgery, Aseer Central Hospital, Abha 62523, Saudi Arabia; ktardi@kfmcity.med.sa; 4 College of Medicine, King Khalid University, Abha 61421, Saudi Arabia; ghalaahmr2@gmail.com (G.S.A.A.); danah.almutib@gmail.com (D.M.A.M.); 5 College of Medicine, Taif University, Taif 26571, Saudi Arabia; razan.v06@gmail.com; 6 College of Medicine, Najran University, Najran 66251, Saudi Arabia; najla.mohamed.2025@gmail.com; 7 Faculty of Dentistry, Al Baha University, Al Baha 65779, Saudi Arabia; abdoulh132@gmail.com

**Keywords:** tinnitus, prevalence, epidemiology, Saudi Arabia, noise exposure, healthcare utilization

## Abstract

Background/Objectives: Tinnitus is a significant global health concern with substantial geographic variation in prevalence. However, data on tinnitus epidemiology in southern Saudi Arabia remain limited, despite emerging evidence suggesting a relatively high regional burden. Therefore, this study aimed to estimate the prevalence of tinnitus and identify its epidemiological determinants among adults in southern Saudi Arabia. Methods: A community-based cross-sectional study was conducted among 501 adults from four regions in southern Saudi Arabia (Jazan, Aseer, Najran, and Al-Baha). Data were collected on sociodemographic characteristics, chronic medical conditions, noise exposure patterns, and clinical features of tinnitus. Multivariable logistic regression analysis was used to identify factors independently associated with tinnitus. Results: The overall tinnitus prevalence was 31.1% (156/501). The mean age of participants was 30 ± 11 years, and 61.7% were female. Bilateral tinnitus was the most common (47.4%). Symptom duration was reported as <3 months in 31.4% of cases and between 3 months and 1 year in another 31.4%. The mean severity score was 3.7 ± 1.9. Most affected individuals (76.9%) did not seek medical care. Primary exacerbating factors included sleep deprivation (41.7%), noise exposure (41.0%), and stress (39.1%). Multivariable analysis identified two significant independent predictors: weekly noise exposure exceeding 5 h (odds ratio [OR] = 2.14; 95% confidence interval [CI]: 1.34–3.42; *p* = 0.001) and higher body mass index (BMI) (OR = 1.06; 95% CI: 1.02–1.10; *p* = 0.005). The most commonly associated underlying conditions were ear infections (25.6%) and hearing loss (24.4%). Conclusions: Tinnitus prevalence in southern Saudi Arabia (31.1%) substantially exceeds global estimates. Noise exposure and elevated BMI are key modifiable factors associated with tinnitus. The substantial healthcare gap, with most affected individuals not seeking treatment, represents a critical public health challenge requiring targeted prevention programs, provider education, and improved access to evidence-based care.

## 1. Introduction

Tinnitus, defined as the perception of sound in the absence of external acoustic stimuli, represents a significant global health challenge affecting millions of individuals worldwide [1]. This auditory condition can substantially impair quality of life, including sleep, concentration, and psychological well-being [2]. Globally, more than 740 million adults experience tinnitus, with over 120 million reporting it as a major problem [1].

Prevalence estimates vary considerably across regions, with a pooled global prevalence of 14.4% (95% confidence interval [CI]: 12.6–16.5%) among adults [1]. Reported rates include 20.7% in Korea, 14.7% across European Union nations, 9.6% in the United States, and 5.17% in Egypt [3,4,5,6]. Such variability likely reflects differences in study methodologies, population characteristics, and environmental factors [7].

The etiology of tinnitus is multifactorial. Established risk factors include noise exposure, hearing loss, aging, and cardiovascular disease [3]. Among these, noise exposure is among the most significant modifiable risk factors [5]. Tinnitus is also strongly associated with sleep disturbances, anxiety, and depression [8].

In Saudi Arabia, emerging evidence suggests that tinnitus prevalence may exceed global averages. National studies report rates ranging from 6.54% [2] to 37.6% [9]. Substantial regional variation has also been observed, with studies indicating higher prevalence in the southern regions: 28.5% in Aseer [10] and 33% in Taif [11]. Despite this burden, a large proportion of affected individuals do not seek medical care; reported treatment-seeking rates range from 39% to 61% [2,9].

The southern region of Saudi Arabia, encompassing Jazan, Aseer, Najran, and Al-Baha, represents a distinct geographic area with unique environmental and occupational characteristics, including elevated agricultural and construction noise exposure, high ambient temperatures that may influence vascular function, and differing patterns of occupational hazard relative to other Saudi regions. Al-Qurashi et al. [12] highlighted significant regional health disparities within the country, underscoring the need for region-specific research. However, comprehensive community-based studies examining tinnitus prevalence, associated factors, and healthcare utilization in southern Saudi Arabia remain limited [12].

Therefore, this study aimed to determine the prevalence of tinnitus among adults in southern Saudi Arabia; identify associated epidemiological determinants and risk factors; characterize patterns of clinical presentation; assess healthcare utilization behaviors; and examine factors that exacerbate tinnitus symptoms. The findings will provide essential baseline data to inform healthcare planning and guide targeted prevention and management strategies for this understudied population.

## 2. Materials and Methods

### 2.1. Study Design and Setting

This cross-sectional study was conducted between 2025 and 2026 in the southern region of Saudi Arabia, including Jazan, Aseer, Najran, and Al-Baha. The study aimed to assess the prevalence, characteristics, and associated factors of tinnitus among adults residing in these regions. A convenience sampling approach combined with online chain referral was employed to recruit participants through digital platforms to ensure broad participation from the targeted regions.

### 2.2. Participants and Sampling

The target population comprised residents of the southern region of Saudi Arabia aged ≥ 18 years during the study period (2025–2026). Inclusion criteria were: 18 years or older, residence in Jazan, Aseer, Najran, or Al-Baha, ability to understand Arabic, and willingness to participate in the study. Exclusion criteria included individuals younger than 18 years, non-Arabic speakers, residents outside the southern region of Saudi Arabia, and those who declined participation.

The sample size was calculated using the Raosoft sample size calculator (http://www.raosoft.com/samplesize.html (accessed on 15 March 2026)). Based on an estimated population of 1,404,997 individuals in the southern region of Saudi Arabia (according to the General Authority for Statistics), a 95% confidence level, 5% margin of error, and an assumed response distribution of 50%, the minimum required sample size was 385 participants. A total of 501 participants were ultimately included, exceeding the minimum requirement and enhancing the study’s statistical power.

Participants were recruited using a non-random convenience sampling technique with online chain-referral (snowball) techniques. The electronic survey was distributed via social media platforms, and participants were encouraged to share the survey link with their networks to increase coverage and participation across the targeted regions.

### 2.3. Data Collection Instrument

Data were collected using a structured, pre-designed questionnaire adapted from a previously published study conducted in Taif, Saudi Arabia [11]. The instrument included items assessing sociodemographic characteristics, medical history, lifestyle factors, and tinnitus-related information, including severity and impact on daily life.

Tinnitus prevalence was assessed using the question:

“Do you experience, or have you recently experienced, continuous or intermittent tinnitus (i.e., hearing internal sounds such as buzzing or ringing in one or both ears)?”

Tinnitus severity was assessed using a Visual Analog Scale (VAS) ranging from 0 to 10, with higher scores indicating greater symptom severity. A score > 7 was considered indicative of severe tinnitus. Participants were instructed to consider their tinnitus experience over the preceding 12 months. Participants reporting only transient or momentary auditory phenomena were not classified as having tinnitus. The four noise exposure variables were operationalized as follows: (1) weekly noise exposure > 5 h: self-reported cumulative noise exposure exceeding 5 h per week from any source; (2) recreational noise exposure: participation in leisure activities involving noise, such as personal audio device use or attendance at loud events; (3) sudden loud sound exposure: any reported exposure to high-intensity impulsive noise such as fireworks, gunshots, or explosions; and (4) occupational noise exposure > 3 months: work-related noise exposure sustained for more than 3 months.

All questionnaire items were translated into Arabic, the native language of the target population. A pilot study involving 30 participants was conducted prior to the main data collection to evaluate clarity, reliability, and feasibility and to identify any potential issues with survey completion. Data from pilot participants were excluded from the final analysis.

### 2.4. Statistical Analysis

Following data collection, all responses were reviewed manually for completeness and accuracy. The collected data were then coded and entered into Microsoft Excel (Microsoft Corporation, Redmond, WA, USA) for preliminary data management. Statistical analyses were conducted using RStudio (version 4.2.3; R Foundation for Statistical Computing, Vienna, Austria).

Descriptive statistics were used to summarize participant characteristics. Continuous variables are presented as means with standard deviations, while categorical variables are reported as frequencies and percentages. Inferential analyses included independent *t*-tests and analysis of variance for normally distributed continuous variables and chi-square tests for categorical variables. Statistical significance was set at *p* < 0.05. Variables for inclusion in the multivariable logistic regression were selected a priori based on clinical relevance and evidence from the tinnitus literature. Multicollinearity was assessed using variance inflation factors (VIF); all binary predictors had VIF values between 1.12 and 2.38, indicating no problematic collinearity. Model fit was evaluated using the Hosmer–Lemeshow test and Nagelkerke R^2^, with results reported alongside the regression table.

### 2.5. Ethical Considerations

The study protocol was reviewed and approved by the Research Ethics Committee of Jazan University (Reference No.: REC-46/12/1553, approval date: 30 May 2025). The study was conducted in accordance with the ethical principles outlined in the Declaration of Helsinki. Participation was voluntary, and electronic informed consent was obtained from all participants prior to survey completion. Participants were assured of anonymity and confidentiality, and no identifying information was collected. All data were used solely for research purposes and were accessible only to the research team.

## 3. Results

### 3.1. Sociodemographic Characteristics of the Study Participants

A total of 501 participants were included in the study. The mean age was 30 ± 11 years. Females comprised 61.7% of the sample, while males accounted for 38.3%. Regarding marital status, 58.9% were single, 37.1% were married, and 4.0% were divorced or widowed. Participants were recruited from four regions in southern Saudi Arabia, with the largest proportion from Jazan (31.1%), followed by Aseer (26.7%), Najran (21.6%), and Al-Baha (20.6%).

Most participants had a university-level education or higher (81.8%); 16.4% had completed high school, and 1.8% had a middle school education or less. The mean body mass index (BMI) was 25 ± 5.5 kg/m^2^. Most participants reported never smoking (86.0%), while 8.4% were former smokers and 5.6% were current smokers. Table 1 presents detailed sociodemographic characteristics.

### 3.2. Chronic Conditions Among Participants

Table 2 summarizes the prevalence of chronic medical conditions among the study participants. Hypertension and depressive disorders were the most frequently reported conditions, each affecting 8.0% of participants, followed by dyslipidemia (7.6%) and diabetes mellitus (7.4%). Less commonly reported conditions included thyroid disorders (2.0%), asthma (1.6%), irritable bowel syndrome (1.2%), and anemia (1.0%). Rarely reported conditions included renal disease (0.6%), celiac disease (0.2%), pelvic inflammatory disease (0.2%), and rheumatoid arthritis (0.2%).

### 3.3. Noise Exposure Characteristics

Table 3 presents noise exposure characteristics among the study population. Overall, 39.1% of participants reported weekly noise exposure exceeding 5 h, while 45.7% reported recreational noise exposure. Exposure to sudden loud sounds, such as fireworks, gunshots, or explosions, was reported by 42.5% of participants. Additionally, 20.8% reported occupational noise exposure lasting longer than 3 months, whereas the majority (79.2%) reported no occupational exposure.

### 3.4. Prevalence and Clinical Characteristics of Tinnitus (Past 12 Months)

The overall prevalence of current or past 12-month tinnitus among the study participants was 31.1% (156/501), while 68.9% reported no tinnitus symptoms. Among those with tinnitus, the most commonly reported presentation was bilateral tinnitus or tinnitus perceived inside the head (47.4%), followed by right-sided tinnitus (29.5%) and left-sided tinnitus (23.1%).

Regarding duration, 31.4% reported tinnitus lasting less than 3 months, and an equal proportion (31.4%) reported symptoms lasting between 3 months and 1 year. Additionally, 22.4% reported tinnitus lasting between 1 and 5 years, and 14.7% reported symptoms persisting for more than 5 years. The mean tinnitus severity score was 3.7 ± 1.9.

Most individuals with tinnitus did not seek medical care (76.9%), while 17.3% had previously received treatment and 5.8% were currently undergoing treatment. The most commonly reported treatment modalities included medication (23.7%), management of an underlying cause (10.9%), behavioral therapy (7.1%), and hearing aids (2.6%).

Among underlying conditions associated with tinnitus, ear infections (25.6%) and hearing loss (24.4%) were the most frequently reported factors, followed by temporomandibular joint disorder (10.9%). Table 4 summarizes these findings.

### 3.5. Distribution of Tinnitus Severity

**Figure 1:**
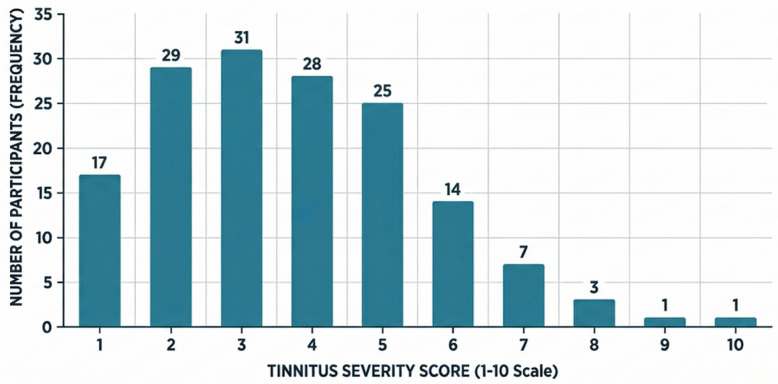
Distribution of Tinnitus Severity Scores among Study Participants (*n* = 156).

Figure 1 presents the distribution of tinnitus severity scores among participants with tinnitus (*n* = 156). The data demonstrates variability in perceived tinnitus severity among affected individuals, with most participants reporting mild-to-moderate symptoms.

### 3.6. Impact of Tinnitus on Daily Life

**Figure 2:**
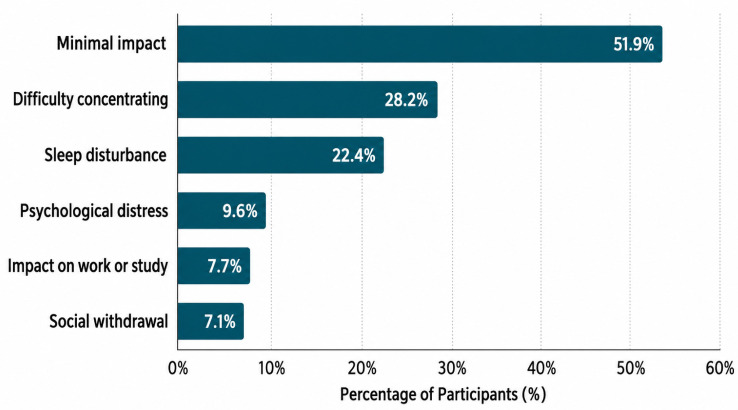
Distribution of the Impact of Tinnitus on the Daily Life of Study Participants (*n* = 156).

Figure 2 illustrates the perceived impact of tinnitus on daily functioning among affected participants. The results show varying degrees of daily life disruption attributable to tinnitus symptoms, highlighting the functional burden experienced by some affected individuals.

### 3.7. Exacerbating Factors of Tinnitus

**Figure 3:**
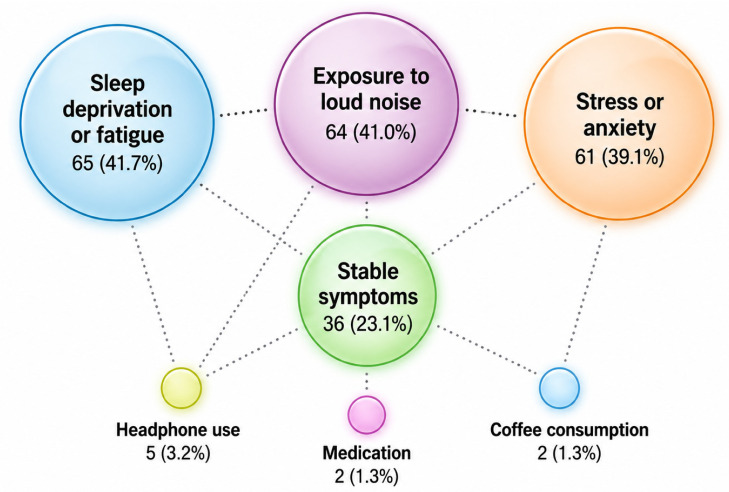
Exacerbating Factors of Tinnitus among Study Participants (*n* = 156). Participants could select multiple responses; percentages do not sum to 100%.

Participants with tinnitus reported several factors that worsened their symptoms (Figure 3). The most frequently reported exacerbating factors included sleep deprivation or fatigue (41.7%), exposure to loud noise (41.0%), and stress or anxiety (39.1%). A notable proportion of participants reported stable symptoms with no identifiable triggers (23.1%). Less commonly reported exacerbating factors included headphone use (3.2%), medication (1.3%), and coffee consumption (1.3%).

### 3.8. Factors Associated with Tinnitus

Multivariable logistic regression analysis was conducted to identify factors independently associated with tinnitus (Table 5). Among the examined variables, BMI and weekly noise exposure exceeding 5 h were significantly associated with tinnitus.

Higher BMI was associated with increased odds of tinnitus (odds ratio [OR] = 1.06; 95% CI: 1.02–1.10, *p* = 0.005). Additionally, participants reporting more than 5 h of weekly noise exposure had more than twice the odds of tinnitus compared with those without such exposure (OR = 2.14; 95% CI: 1.34–3.42, *p* = 0.001).

Other variables, including age, sex, marital status, residence, education level, smoking status, hypertension, diabetes mellitus, depressive disorder, recreational noise exposure, exposure to sudden loud sounds, and occupational noise exposure, were not significantly associated with tinnitus in the adjusted model. All binary predictors had VIF values between 1.12 and 2.38, indicating no problematic collinearity. The moderate correlation between age and BMI (r = 0.37) is consistent with expected population-level patterns and did not compromise model integrity. Model fit was satisfactory, as indicated by the Hosmer–Lemeshow test (χ^2^ = 4.865, df = 8, *p* = 0.772) and Nagelkerke R^2^ = 0.139.

## 4. Discussion

### 4.1. Prevalence of Tinnitus in Southern Saudi Arabia

This study identified a 31.1% tinnitus prevalence among adults surveyed in southern Saudi Arabia, particularly among younger, digitally engaged individuals, representing one of the highest documented rates reported both regionally and internationally. This finding aligns closely with recent regional findings: Musleh et al. [10] found a prevalence of 28.5% among health science students in Aseer, while Fageeh et al. [11] reported a 33% prevalence in Taif. The consistency of these results across independent studies conducted in different southern provinces suggests a genuine regional elevation rather than study-specific bias or methodological artifact. It should be noted that our estimate includes acute cases (symptom duration <3 months, representing 31.4% of tinnitus cases), which may not be captured in studies applying a minimum duration criterion; the true prevalence of persistent tinnitus in this population may therefore be somewhat lower.

These prevalence estimates substantially exceed both national averages and international benchmarks. Alanazi (2024) reported a 6.54% national prevalence based on face-to-face interviews with 4860 adults, whereas Alkahtani et al. (2024) documented a markedly higher prevalence of 37.6% in a larger national sample of 4416 participants [2,9]. The substantial variation between national studies likely reflects methodological differences in tinnitus assessment, underscoring persistent challenges in measurement standardization [7].

International comparisons reveal our findings exceed most global estimates. Jarach et al. (2022) reported a pooled global prevalence of 14.4% (95% CI: 12.6–16.5%) in a comprehensive systematic review of 113 studies [1]. Reported regional estimates included: 20.7% in Korea [3], 14.5% in China [13], 10.4% in Guangdong Province [14], 9.6% in the United States [5], 6.7% in India [15], 5.17% in Egypt [6], and 14.7% across European Union nations [4]. The markedly higher prevalence in southern Saudi Arabia suggests region-specific contributing factors, including environmental conditions, occupational exposures, or genetic predisposition.

### 4.2. Epidemiological Determinants

#### 4.2.1. Noise Exposure: The Primary Associated Factor

Weekly noise exposure exceeding 5 h emerged as the strongest predictor of tinnitus (OR = 2.14; 95% CI: 1.34–3.42; *p* = 0.001), supporting regional findings by Fageeh et al., who also reported a significant association between prolonged noise exposure and tinnitus in Taif (*p* = 0.000) [11]. This consistency across multiple studies from southern Saudi Arabia identifies noise exposure as a key modifiable associated factor in this population.

The high prevalence of various noise exposures in our study population is concerning. Overall, 39.1% of participants reported weekly exposure exceeding 5 h, 45.7% reported recreational noise exposure, 42.5% experienced sudden loud sound exposure, and 20.8% reported occupational noise exposure lasting longer than 3 months. This pattern may reflect regional occupational characteristics, recreational behaviors, or environmental factors specific to southern Saudi Arabia.

Our findings are consistent with broader international evidence. Haji et al. reported that 22.7% of young adults aged 15–25 years in Makkah developed noise-induced tinnitus, with continuous tinnitus significantly more prevalent in the noise-exposed group (*p* = 0.037) [16]. Similarly, Bhatt et al. demonstrated that years of work-related noise exposure correlated with increasing tinnitus prevalence in the United States (r = 0.13; 95% CI: 0.10–0.16) [5]. In eastern Saudi Arabia, Ahmed et al. documented significant associations between occupational noise exposure and hearing loss, suggesting that noise-related auditory disorders may be widespread across the kingdom [17].

Based on the broader mechanistic literature, proposed pathophysiological mechanisms include cochlear hair cell damage, disrupted neural synchrony within auditory pathways, and maladaptive neuroplastic changes in both central auditory and limbic systems [18], though these were not directly assessed in the present study. Chronic noise exposure may result in permanent threshold shifts and central auditory processing changes that manifest as tinnitus even in individuals with normal audiometric thresholds.

#### 4.2.2. Body Mass Index: A Noteworthy Regional Finding

The significant association between higher BMI and tinnitus (OR = 1.06; 95% CI: 1.02–1.10; *p* = 0.005) represents a noteworthy finding consistent with emerging evidence linking metabolic risk factors to auditory outcomes. Although the odds ratio appears modest on a per-unit basis, it translates to a clinically meaningful dose–response increase in risk. While residual confounding from unmeasured variables such as dietary patterns and physical activity cannot be excluded, the statistical precision of this association supports a biologically plausible rather than spurious finding.

This finding is consistent with regional evidence linking metabolic and cardiovascular risk factors to tinnitus. In southern Saudi Arabia, Musleh et al. (2022) reported a significant association between hyperlipidemia and tinnitus, with elevated lipid profiles associated with approximately a twofold increase in risk (*p* < 0.05) [19]. Similarly, Fageeh et al. (2025) identified a significant association with hypertension (*p* = 0.023), supporting a role for cardiovascular mechanisms in tinnitus pathophysiology [11]. Internationally, Kim et al. (2015) also reported an association between tinnitus and hyperlipidemia in a Korean population [3].

Several biological mechanisms may explain these associations. Obesity-related systemic inflammation may impair cochlear function through inflammatory mediators. In addition, obesity-associated cardiovascular changes, including hypertension and impaired vascular regulation, may compromise cochlear microcirculation. Metabolic syndrome components, including insulin resistance and dyslipidemia, may further disrupt inner ear metabolic homeostasis and auditory function. Given the mean BMI of 25 ± 5.5 kg/m^2^ in the study population, metabolic dysregulation may significantly contribute to the elevated regional tinnitus prevalence.

#### 4.2.3. Demographic and Chronic Disease Factors

Contrary to many international studies, age and sex were not significantly associated with tinnitus in the adjusted model. Jarach et al. (2022) reported that tinnitus prevalence increases significantly with age (9.7% among individuals aged 18–44 years, 13.7% in those aged 45–64 years, and 23.6% in those aged ≥65 years), while no significant sex differences were observed [1]. The absence of an age association in the present study most likely reflects the relatively young and age-homogeneous study population (mean age: 30 ± 11 years). This age compression limits the statistical power to detect age gradients that would be evident in a more broadly representative sample. Consistent with this interpretation, Musleh et al. (2020) similarly reported no significant association between age and tinnitus among young students in Aseer [10].

Although individual chronic conditions did not reach statistical significance in our multivariable model, their prevalence patterns provide insights. Hypertension and depressive disorders were the most frequently reported comorbidities (8.0% each), followed by dyslipidemia (7.6%) and diabetes mellitus (7.4%). The lack of significant associations in the present study contrasts with prior international evidence. For example, Kim et al. (2015) identified significant associations between tinnitus and hyperlipidemia, depression, and thyroid disease, while regional findings by Fageeh et al. (2025) also documented significant associations with hypertension and depression [3,11].

### 4.3. Clinical Characteristics and Healthcare Utilization

#### 4.3.1. Tinnitus Presentation and Severity

Bilateral tinnitus or tinnitus perceived inside the head predominated (47.4%), closely aligning with Alanazi (2024), who reported a 51.8% prevalence of bilateral tinnitus nationally, and Musleh et al. (2020), who observed a similar 51.8% rate among students in Aseer [2,10]. This consistency across studies suggests that bilateral presentation may be a characteristic feature of tinnitus in Saudi populations, with potential implications for understanding its underlying etiology and informing clinical management strategies.

The temporal distribution of symptoms demonstrates distinct clinical patterns. Approximately 31.4% of participants reported symptoms lasting less than 3 months, 31.4% between 3 months and 1 year, 22.4% between 1 and 5 years, and 14.7% persisting for more than 5 years. This distribution suggests that a substantial proportion of cases are acute or subacute, which may be more amenable to intervention compared with chronic presentations. Rammal et al. (2024) similarly reported that 24.2% of patients with chronic tinnitus experienced symptoms exceeding 2 years, noting that longer symptom duration is associated with increased sleep disturbance and greater functional impairment [20].

The mean tinnitus severity score (VAS) of 3.7 ± 1.9 indicates a mild-to-moderate symptom burden, with most affected individuals rating their tinnitus between 1 and 4. This is clinically consistent with the finding that 76.9% did not seek care: mild perceived severity reduces the perceived need for medical consultation, suggesting that low treatment-seeking partly reflects the intrinsic severity distribution of the sample rather than purely systemic barriers. This finding is broadly consistent with Alanazi (2024) and Alkahtani et al. (2024), who reported a mean Tinnitus Handicap Inventory score of 16.7, corresponding to a mild level of perceived handicap [2,9].

#### 4.3.2. Healthcare Utilization: A Critical Gap

The finding that 76.9% of individuals with tinnitus did not seek medical care represents a critical healthcare gap with significant public health implications. This pattern is consistent with prior studies in Saudi Arabia, including reports that approximately 61% of individuals received no tinnitus treatment and that only 39.7% sought medical support. The consistency of these findings across multiple Saudi studies suggests that the gap is driven by systemic barriers rather than study-specific variation [2,9].

The reasons underlying this care gap were not directly assessed; therefore, the following should be regarded as hypothesis-generating rather than established explanations. Plausible contributing factors based on the broader literature include cultural perceptions of tinnitus as a self-limiting condition, limited patient and provider awareness of treatment options, and constraints in specialist access. Alsanosi (2011) emphasized the need for improved tinnitus-related services and standardized assessment tools for Saudi healthcare settings [21]. Future research should directly assess barriers to care in this population.

Among individuals who did seek care, medication was most frequently reported (23.7%), followed by treatment targeting underlying causes (10.9%), behavioral therapy (7.1%), and hearing aid use (2.6%). This distribution contrasts with international clinical guidelines, which increasingly prioritize cognitive behavioral therapy, sound therapy, and tinnitus retraining therapy as first-line interventions. Similarly, Alanazi (2024) reported similar patterns with medications being the most frequently discussed management option (45.4%), whereas evidence-based interventions such as hearing aids (9.2%) and masking devices were less commonly utilized [2].

#### 4.3.3. Associated Medical Conditions and Exacerbating Factors

Ear infections (25.6%) and hearing loss (24.4%) were the most frequently reported underlying conditions, providing important etiological insights. These findings are consistent with Alqarny et al. (2021), who reported that tinnitus was associated with 43.1% of hearing loss cases in southern Saudi Arabia [22]. The high prevalence of ear infections may reflect regional environmental conditions, healthcare access patterns, or genetic predisposition to otologic infections.

Temporomandibular joint (TMJ) disorder (10.9%) was identified as another significant associated factor. This is consistent with Khedr et al. (2010), who demonstrated a significant correlation between tinnitus severity and TMJ pain in Egypt [6]. Collectively, these findings support the multifactorial etiology of tinnitus and may reflect shared neural mechanisms, muscle tension effects, or common stress-related mechanisms.

Sleep deprivation or fatigue (41.7%), noise exposure (41.0%), and stress or anxiety (39.1%) were identified as primary exacerbating factors. These findings strongly align with Rammal et al. (2024), who found 62.7% of participants experienced insomnia related to tinnitus, suggesting a bidirectional relationship in which tinnitus disrupts sleep and sleep deprivation, in turn, exacerbates symptoms [20]. Similarly, Musleh et al. (2024) reported substantial impacts of tinnitus in the southern region, with tinnitus affecting concentration (37.42%), hearing (36.81%), and sleep (36.81%), as well as emotional effects such as anger (47.85%) and anxiety (43.56%) [23].

### 4.4. Strengths and Limitations

This study has several notable strengths, including a relatively large sample size (*n* = 501) drawn from four regions in southern Saudi Arabia, a comprehensive assessment of multiple associated factors, and the use of multivariable logistic regression to adjust for confounding. The validity of the findings is further supported by consistency with prior regional studies. Specifically, the observed tinnitus prevalence of 31.1% closely aligns with reports from Aseer (28.5%) [10] and Taif (33%) [11], reinforcing the reliability of the estimates.

However, several important limitations must be acknowledged. The cross-sectional design precludes causal inference. The predominance of younger, female, and university-educated participants reflects the characteristics of digitally recruited convenience samples and may limit generalizability to the broader adult population. The absence of a formal minimum duration criterion in the tinnitus case definition may have resulted in inclusion of transient episodes. The absence of objective audiometric assessment precludes verification of self-reported hearing loss and limits mechanistic interpretation; pathophysiological statements are drawn from the broader literature and were not directly assessed in this study. The reliance on self-reported tinnitus and noise exposure data may introduce recall and reporting bias [7]. Furthermore, the geographic focus on southern Saudi Arabia may limit generalizability to other regions, and potential unmeasured confounders including genetic predisposition, medication use, and socioeconomic status may have influenced observed associations.

### 4.5. Clinical and Public Health Implications

The high prevalence of tinnitus (31.1%) coupled with low healthcare utilization (76.9% not seeking care) suggests substantial unmet clinical needs. The strong association between noise exposure and tinnitus (OR = 2.14) suggests that targeted prevention strategies could significantly reduce the tinnitus burden through hearing conservation initiatives, enforcement of workplace noise regulations, and public awareness campaigns promoting hearing protection. The association with BMI (OR = 1.06) indicates that metabolic health interventions may provide additional auditory benefits, supporting integrated approaches that address both cardiovascular and auditory health outcomes.

Healthcare system responses should prioritize strengthening primary care providers’ capacity to recognize and manage tinnitus, expanding access to specialized audiology services, and implementing standardized assessment protocols that emphasize evidence-based treatments over medications. The observed predominance of medication-based management (23.7%), despite the established effectiveness of interventions such as cognitive behavioral therapy, highlights the urgent need for improved provider education. Alanazi (2024) similarly highlighted the necessity of standardized practice guidelines and improved access to evidence-based interventions [2].

Future research should focus on longitudinal study designs to clarify causal relationships between identified risk factors and tinnitus onset and progression. Studies should also incorporate objective audiometric assessments and standardized severity measures such as the Tinnitus Handicap Inventory [9]. Further investigation into the biological mechanisms linking BMI and tinnitus is warranted to inform potential therapeutic approaches. In addition, research examining barriers to healthcare access could guide service delivery improvements, while genetic studies may help explain the higher regional prevalence compared with international estimates. Finally, implementation research is needed to evaluate effective models for integrating tinnitus care into existing healthcare systems and to address the substantial gap between prevalence and treatment uptake.

## 5. Conclusions

This study demonstrates a high prevalence of tinnitus (31.1%) among adults surveyed in southern Saudi Arabia, particularly younger, digitally engaged individuals, with noise exposure and BMI identified as key modifiable factors. The consistent findings across regional studies (28.5–33%) suggest genuine geographic disparities that warrant targeted public health interventions, particularly given the substantial care gap, with most affected individuals (76.9%) not seeking treatment. These findings underscore the importance of preventive strategies, including hearing conservation and metabolic health interventions, as well as the need to improve provider awareness of evidence-based management beyond medication use. Future research should prioritize longitudinal study designs, incorporate objective clinical assessments, and evaluate implementation strategies. In addition, developing region-specific guidelines and public awareness initiatives will be essential to addressing the treatment gap and improving tinnitus care outcomes.

## Figures and Tables

**Table 1:** Sociodemographic characteristics of the study participants (*n* = 501).

Characteristics	Mean ± SD/Frequency (%)
**Age (years)**	30 ± 11
**Sex**	
Female	309 (61.7%)
Male	192 (38.3%)
**Marital status**	
Single	295 (58.9%)
Married	186 (37.1%)
Divorced/Widowed	20 (4.0%)
**Residence**	
Jazan	156 (31.1%)
Aseer	134 (26.7%)
Najran	108 (21.6%)
Al-Baha	103 (20.6%)
**Education level**	
University or higher	410 (81.8%)
High school	82 (16.4%)
Middle school or less	9 (1.8%)
**BMI (kg/m^2^)**	25 ± 5.5
**Smoking status**	
Never	431 (86.0%)
Ex-smoker	42 (8.4%)
Current smoker	28 (5.6%)

Abbreviations: *n*: sample size, SD: standard deviation, BMI: body mass index.

**Table 2:** Chronic conditions among the study participants (*n* = 501).

Characteristics	Frequency (%)
**Chronic conditions**	
Hypertension (HTN)	40 (8.0%)
Diabetes mellitus (DM)	37 (7.4%)
Dyslipidemia	38 (7.6%)
Depressive disorder	40 (8.0%)
Asthma	8 (1.6%)
Thyroid disorder	10 (2.0%)
Renal condition	3 (0.6%)
Anemia	5 (1.0%)
Irritable bowel syndrome (IBS)	6 (1.2%)
Celiac disease	1 (0.2%)
Pelvic inflammatory disease (PID)	1 (0.2%)
Rheumatoid arthritis (RA)	1 (0.2%)

Abbreviations: HTN: hypertension, DM: diabetes mellitus, IBS: irritable bowel syndrome, PID: pelvic inflammatory disease, RA: rheumatoid arthritis.

**Table 3:** Noise exposure characteristics among the study participants (*n* = 501).

Characteristics	Frequency (%)
**Weekly noise exposure (>5 h)**	
No	305 (60.9%)
Yes	196 (39.1%)
**Recreational noise exposure**	
No	272 (54.3%)
Yes	229 (45.7%)
**Exposure to sudden loud sounds (e.g., fireworks, gunshots, explosions)**	
No	288 (57.5%)
Yes	213 (42.5%)
**Occupational noise exposure (>3 months)**	
No	397 (79.2%)
Yes	104 (20.8%)

**Table 4:** Prevalence and characteristics of tinnitus among the study participants.

Characteristics	Mean ± SD/Frequency (%)
**Current or past 12-month tinnitus (unilateral or bilateral) (*n* = 501)**	
No	345 (68.9%)
Yes	156 (31.1%)
**Side of tinnitus (*n* = 156)**	
Both ears/inside the head	74 (47.4%)
Right ear only	46 (29.5%)
Left ear only	36 (23.1%)
**Duration of tinnitus (*n* = 156)**	
Less than 3 months	49 (31.4%)
3 months to 1 year	49 (31.4%)
1–5 years	35 (22.4%)
>5 years	23 (14.7%)
**Tinnitus severity score (VAS) (*n* = 156)**	3.7 ± 1.9
**Sought medical or professional care for tinnitus (*n* = 156)**	
No, did not seek treatment	120 (76.9%)
Previously received treatment	27 (17.3%)
Currently receiving treatment	9 (5.8%)
Type of medical care received *	
Medication	37 (23.7%)
Treatment of the underlying cause	17 (10.9%)
Behavioral treatment	11 (7.1%)
Hearing aid	4 (2.6%)
Ear cleaning	2 (1.3%)
Underlying medical conditions associated with tinnitus *	
Ear infection	40 (25.6%)
Hearing loss	38 (24.4%)
Temporomandibular joint disorder (TMJ)	17 (10.9%)
Head or neck injury	3 (1.9%)
Tympanic membrane perforation	1 (0.6%)
Otosclerosis	1 (0.6%)

* indicates multiple responses.

**Table 5:** Multivariable logistic regression analysis of factors associated with tinnitus among the study participants (*n* = 501).

	Tinnitus		
Predictors	OR	95% CI	*p*
**Age (years)**	0.99	0.96–1.03	0.706
**Sex** (reference: [Female])			
[Male]	0.66	0.38–1.13	0.132
**Marital status** (reference: [Single])			
[Divorced/Widowed]	0.78	0.24–2.40	0.675
[Married]	0.64	0.32–1.27	0.209
**Residence** (reference: [Jazan])			
[Al-Baha]	1.11	0.61–2.00	0.735
[Aseer]	1.32	0.76–2.27	0.322
[Najran]	0.64	0.33–1.22	0.181
**Education level** (reference: [Middle school or less])			
[High school]	1.10	0.22–8.42	0.914
[University or higher]	1.72	0.37–12.60	0.526
**BMI (kg/m^2^)**	1.06	1.02–1.10	0.005
**Smoking status** (reference: [Current])			
[Ex-smoker]	1.02	0.33–3.20	0.974
[Never]	1.03	0.40–2.81	0.955
**Hypertension** (reference: [No])			
[Yes]	1.13	0.50–2.49	0.759
**Diabetes mellitus** (reference: [No])			
[Yes]	1.32	0.54–3.05	0.527
**Depressive disorder** (reference: [No])			
[Yes]	1.30	0.62–2.70	0.484
**Weekly noise exposure (>5 h)** (reference: [No])			
[Yes]	2.14	1.34–3.42	0.001
**Recreational noise exposure** (reference: [No])			
[Yes]	1.36	0.86–2.17	0.190
**Exposure to sudden loud sounds** (reference: [No])			
[Yes]	1.16	0.75–1.78	0.503
**Occupational noise exposure (>3 months)** (reference: [No])			
[Yes]	1.30	0.76–2.21	0.337

## Data Availability

The data supporting the findings of this study are not publicly available due to privacy and ethical restrictions but are available from the corresponding author upon reasonable request.

## Abbreviations

The following abbreviations are used in this manuscript:

BMI	Body Mass Index
CI	Confidence Interval
DM	Diabetes Mellitus
HTN	Hypertension
IBS	Irritable Bowel Syndrome
PID	Pelvic Inflammatory Disease
RA	Rheumatoid Arthritis
SD	Standard Deviation
TMJ	Temporomandibular Joint
VAS	Visual Analog Scale
OR	Odds Ratio
